# Sleep duration and resting fMRI functional connectivity: examination of short sleepers with and without perceived daytime dysfunction

**DOI:** 10.1002/brb3.576

**Published:** 2016-09-15

**Authors:** Brian J. Curtis, Paula G. Williams, Christopher R. Jones, Jeffrey S. Anderson

**Affiliations:** ^1^ Department of PsychologyUniversity of UtahSalt Lake CityUTUSA; ^2^ Department of NeurologyUniversity of UtahSalt Lake CityUTUSA; ^3^ Department of RadiologyUniversity of UtahSalt Lake CityUTUSA

**Keywords:** environmental stimulation, fatigue, resting functional connectivity, sleep duration

## Abstract

**Background:**

Approximately 30% of the U.S. population reports recurrent short sleep; however, perceived sleep need varies widely among individuals. Some “habitual short sleepers” routinely sleep 4–6 hr/night without self‐reported adverse consequences. Identifying neural mechanisms underlying individual differences in perceived sleep‐related dysfunction has important implications for understanding associations between sleep duration and health.

**Method:**

This study utilized data from 839 subjects of the Human Connectome Project to examine resting functional connectivity associations with self‐reported short sleep duration, as well as differences between short sleepers with versus without reported dysfunction. Functional connectivity was analyzed using a parcellation covering the cortical, subcortical, and cerebellar gray matter at 5 mm resolution.

**Results:**

Self‐reported sleep duration predicts one of the primary patterns of intersubject variance in resting functional connectivity. Compared to conventional sleepers, both short sleeper subtypes exhibited resting fMRI (R‐fMRI) signatures consistent with diminished wakefulness, potentially indicating inaccurate perception of functionality among those denying dysfunction. Short sleepers denying dysfunction exhibited increased connectivity between sensory cortices and bilateral amygdala and hippocampus, suggesting that efficient sleep‐related memory consolidation may partly explain individual differences in perceived daytime dysfunction.

**Conclusions:**

Overall, current findings indicate that R‐fMRI investigations should include assessment of average sleep duration during the prior month. Furthermore, short sleeper subtype findings provide a candidate neural mechanism underlying differences in perceived daytime impairment associated with short sleep duration.

## 1 Introduction

The minimum amount of sleep humans need to thrive remains controversial. The National Sleep Foundation recommends that young adults (ages 18–25) and adults (ages 26–64) sleep 7 to 9 hr each day (Hirshkowitz, Whiton, Albert, Alessi, & Bruni, 2015). Short sleep duration (i.e., ≤6 hr per night) has been associated with diminished cognitive performance (e.g., Lim & Dinges, 2010; Waters & Bucks, 2011), mood disturbance (e.g., Watson et al., 2014; Wulff, Gatti, Wettstein, & Foster, 2010), weight gain (e.g., Grandner, Chakravorty, Perlis, Oliver, & Gurubhagavatula, 2014; Markwald et al., 2013), inflammation (e.g., Grandner, Sands‐Lincoln, Pak, & Garland, 2013; Kurien, Chong, Ptáček, & Fu, 2013), and increased all‐cause mortality risk (e.g., Grandner, Hale, Moore, & Patel, 2010). These associations are concerning, as 30% of adult workers in the United States report regularly sleeping 6 hr or less, on average, in a 24‐hour period (Luckhaupt, Tak, & Calvert, 2010) – classifying these individuals as “habitual short sleepers” (Grandner, Patel, Gehrman, Perlis, & Pack, 2010).

Prior research suggests that habitual short sleepers are a heterogeneous group, with subtypes that vary in behavioral activation patterns and the extent to which there is perceived daytime dysfunction in relation to short sleep. Short sleepers denying dysfunction have been described as ambitious and extraverted with high levels of behavioral activation (Hartmann, Baekeland, & Zwilling, 1972). In particular, a primary difference between medium‐length sleepers (7.1–7.8 hr of sleep) and short sleepers denying dysfunction (5.3–6.3 hr of sleep) is evidence of “subclinical hypomanic symptoms” (Monk, Buysse, Welsh, Kennedy, & Rose, 2001) and sustained elevations in waking behavioral drive and environmental stimulation seeking (Curtis, Brewer, & Jones, 2011; He et al., 2009). Evidence suggests there are genetic or “natural” short sleepers denying dysfunction with mutations that recapitulate their short sleep phenotype in transgenic animals (e.g., hDEC2‐P385R; He et al., 2009). However, the prevalence of genetically determined short sleep in humans remains unknown. It is plausible that short sleepers denying dysfunction also comprise individuals who may be chronically sleep deprived and would function more optimally with increased sleep. In contrast, short sleepers who report sleep‐related dysfunction are characterized as high in behavioral inhibition, propensity to anxiety (i.e., neuroticism), and hyperarousal (Dorsey & Bootzin, 1997; Duggan, Friedman, McDevitt, & Mednick, 2014). Similar to short sleepers denying dysfunction, short sleepers reporting dysfunction likely comprise a heterogeneous subtype (e.g., individuals with *insomnia with objectively short sleep duration* or *insufficient sleep syndrome* [American Academy of Sleep Medicine, 2014]). The neural mechanisms underlying differences in perceived daytime dysfunction in subtypes of short sleepers remains unknown and, importantly, epidemiological studies of short sleep have not differentiated those that report versus deny having difficulty maintaining wakefulness and functionality (i.e., report vs. deny “daytime dysfunction”).

Short sleep duration figures prominently in a vigilance model of affective disorders (Hegerl & Hensch, 2014) which posits that vigilance regulation (tonic neurophysiologic arousal) is a key neurobiological mechanism. Specifically, behaviors characteristic of mania and ADHD are thought to reflect autoregulatory attempts to stabilize vigilance by creating a stimulating environment, overriding the physiological need to seek sleep. If environmental stimulation is removed, the model predicts a rapid transition from wakefulness to sleep, consistent with historical findings in patients exhibiting mania (e.g., Van Sweden, 1986). Given the presumed heterogeneity of short sleeper subtypes, it remains unknown whether those that deny daytime dysfunction will exhibit diminished wakefulness under low environmental stimulation (e.g., resting‐state functional magnetic resonance imaging (R‐fMRI) assessment), even with instruction to maintain wakefulness.

Prior research suggests the transition from wakefulness to sleep has a distinct neural “signature.” Using combined electroencephalography (EEG) and R‐fMRI, Horovitz and colleagues demonstrate that blood‐oxygen‐level‐dependent (BOLD) signals increase in sensory areas of the brain – particularly in visual, motor, and primary auditory cortices – as humans transition from resting wakefulness to sleep (Horovitz et al., 2008). This R‐fMRI signature is reproducible across laboratories (Davis, Tagliazucchi, Jovicich, Laufs, & Hasson, 2016; Larson‐Prior et al., 2009; Tagliazucchi & Laufs, 2014) and can be used as “a surrogate indicator of wakefulness when EEG is not available” (p. 680 Horovitz et al., 2008). Using combined EEG and R‐fMRI, Tagliazucchi and Laufs found one third of 71 participants fell asleep within four minutes of R‐fMRI assessment, with increased BOLD signals in motor and sensory cortices during the transition from wakefulness to stage 1 and stage 2 sleep (Tagliazucchi & Laufs, 2014). The authors developed a support vector machine classifier to determine how many subjects fell asleep during R‐fMRI using the 1000 Functional Connectomes Project database (Biswal et al., 2010) – finding approximately one third of 1,147 subjects fell asleep within 3 min (Tagliazucchi & Laufs, 2014). Whether subjects with difficulty maintaining wakefulness during R‐fMRI were largely comprised of short sleepers or conventional sleepers is currently unknown, as sleep durations were not reported (Tagliazucchi & Laufs, 2014).

Given the prevalence of self‐reported short sleep in the general population (Luckhaupt et al., 2010), examining associations between sleep duration and resting‐state functional connectivity has broad implications for the interpretation of the growing body of fMRI research. Furthermore, examining short sleeper subgroups that differ in reports of dysfunction may provide insight into the neural mechanisms underlying perceived need for sleep. In addition, prior research suggests that those denying daytime dysfunction may, without environmental stimulation, have difficulty maintaining wakefulness (Hegerl & Hensch, 2014) – such as in R‐fMRI assessment. Although factors such as socioeconomic status, occupation, and comorbid health issues may contribute to individual differences in habitual sleep duration (e.g., Ertel, Berkman, & Buxton, 2011; Grandner, Jackson, Pak, & Gehrman, 2012), examining the various factors that contribute to short sleep duration was beyond the scope of the present investigation. Here, using analysis of R‐fMRI functional connectivity in the Human Connectome Project database (Van Essen et al., 2013), we first examined associations with self‐reported sleep duration. Next, brain functional connectivity differences between short sleeper subtypes (i.e., ≤6 hr/night, reporting vs. denying daytime dysfunction) were examined.

## 2 Materials and Methods

We analyzed data from 839 subjects from the Human Connectome Project 900 Subjects Release. Multiband BOLD resting state data from this release (Feinberg et al., 2010; Glasser et al., 2013; Griffanti et al., 2014; Jesmanowicz, Nencka, Li, & Hyde, 2011; Moeller et al., 2010; Setsompop et al., 2012; Van Essen et al., 2013) consisted of FIX ICA cleaned BOLD resting state data (Griffanti et al., 2014). High spatial resolution functional connectivity data was examined in 475 subjects (S500 Release), and regional functional connectivity measurements were obtained in 839 subjects (S900 Release).

### 2.1 Brain parcellation

Functional connectivity was analyzed using a parcellation covering the cortical, subcortical, and cerebellar gray matter at 5 mm resolution. To construct the parcellation, a gray matter mask was compiled from skull stripped BOLD images for 475 subjects (S500 Release) showing voxels where an a priori gray matter mask (grey.nii, SPM 12b) were inside the brain for 95% of subjects. This image was parcellated into 6,923 nonoverlapping 5‐mm diameter ROIs covering the cortical and subcortical gray matter as follows. Each voxel was tested in sequence beginning with the inferior left voxel in the cerebellum. If a voxel was greater than 5 mm distant to voxels already selected, then this voxel was included in the set of ROI center coordinates. When all voxels had been tested, 6,923 voxels remained, and gray matter voxels were parcellated based on which of the 6,923 center coordinates was closest to a given voxel.

### 2.2 Functional connectivity analyses

Fisher transformed correlation coefficients representing functional connectivity were extracted for each pair of 6,923 × 6,923 ROIs separately for each of the four 15‐minute resting state sequences for each subject. This resulted in 23,960,503 “connections” for each resting state sequence analyzed. This connection matrix was averaged for each subject over the four sequences for that subject to obtain a single matrix for each subject. All four runs were used for each subject to maximize the reliability of metrics for each subject, given demonstrated improvements in reliability with aggregate imaging time (Shah, Cramer, Ferguson, Birn, & Anderson, 2016).

**Figure 1:**
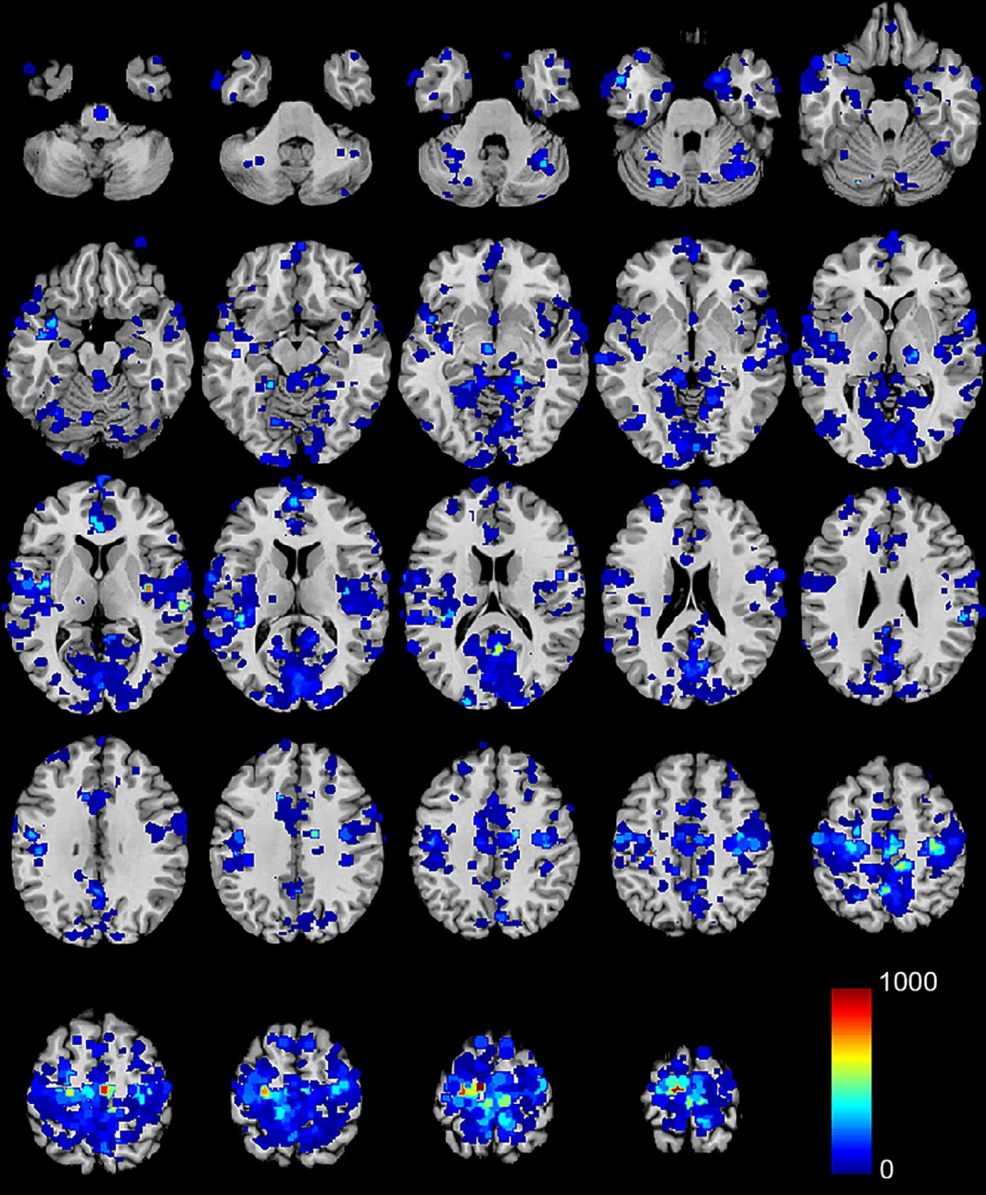
Regions for which functional connectivity significantly covaried with reported sleep duration. Color scale shows for each ROI how many of the 6,922 connections to other ROIs were correlated with sleep duration. The top 50% of ROIs are colored

Reported average sleep duration was determined for each subject using the Pittsburg Sleep Quality Index (Buysse, Reynolds, Monk, Berman, & Kupfer, 1989) included with the Human Connectome Project 900 subject release. For the 839 subjects included in the analysis, reported average sleep duration in the prior month ranged from 2.5 to 12 hr (mean 6.82 h ± 1.15 h *SD*). Pearson correlation coefficient was calculated across subjects between sleep duration and functional connectivity for each pair of 6,923 × 6,923 ROIs in the gray matter. Connections were considered significantly correlated with sleep duration if they satisfied an acceptable false discovery rate of *q* < .05 across all connections. 65,963 connections met these criteria. To establish which regions most frequently participated in connections significantly covarying with sleep duration, significant connections were tabulated by ROI for the 475 subjects of the S500 release and displayed as a brain image (Fig. 1).

**Figure 2:**
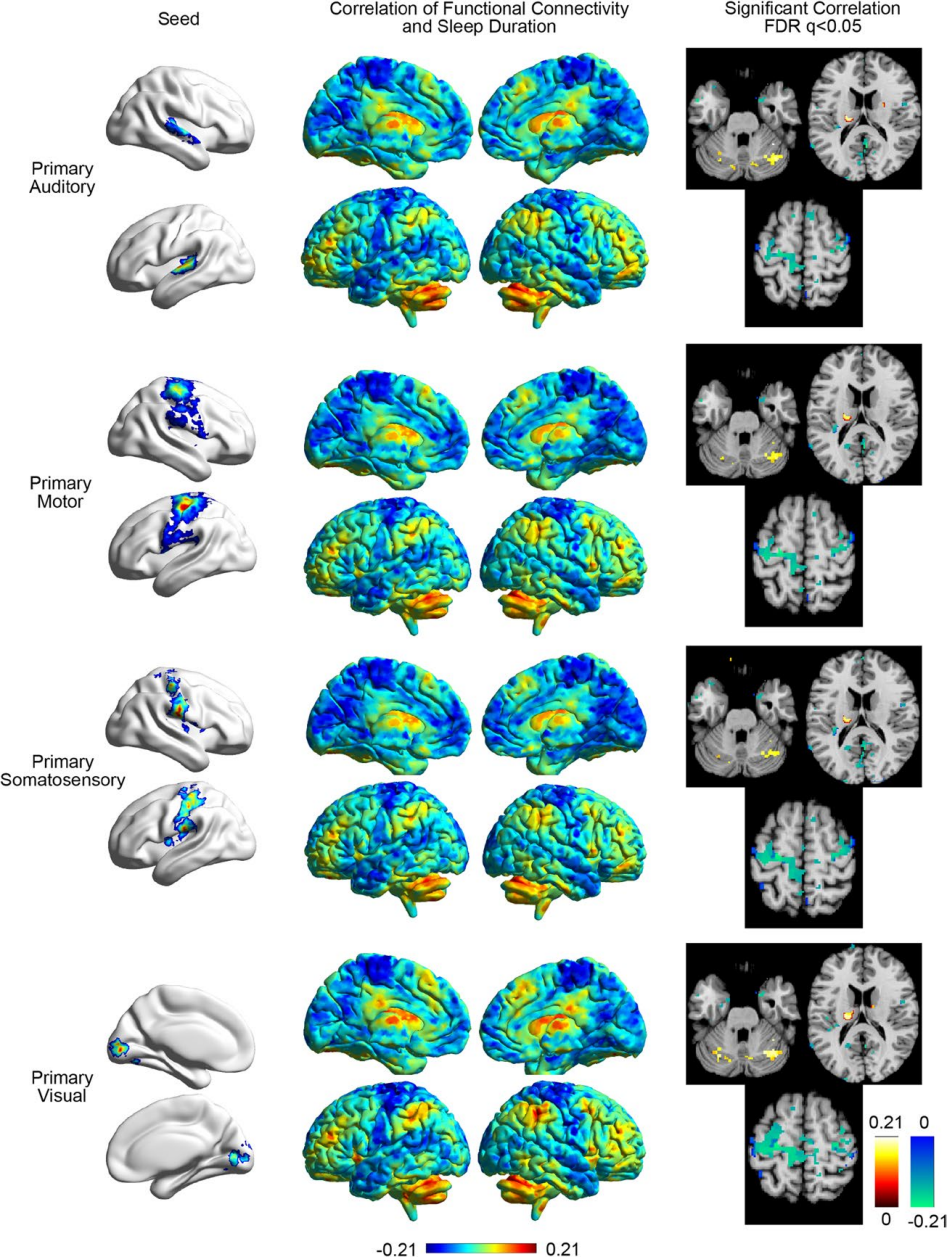
Correlation of functional connectivity to sensory/motor cortex and sleep duration. Left: Seeds used for auditory, motor, somatosensory, and visual cortex. Middle: Correlation of functional connectivity to the seed and other regions in the brain. Right: Slices showing significant covariation between functional connectivity to the seed and sleep duration. Slices are in neurological format with subject left on image left. Slice locations: MNI *z *= −25, *z *= 20, *z *= 65

Given that areas most commonly represented among significant connections included sensory and motor cortices, these regions were selected for further analysis. To define seed regions associated with auditory, somatosensory, visual, and motor cortex, we took advantage of the NeuroSynth database (Yarkoni, Poldrack, Nichols, Van Essen, & Wager, 2011). We entered the terms “primary auditory,” “primary somatosensory,” “primary motor,” and “primary visual” into the database and obtained false discovery rate corrected forward inference maps for each term representing voxels significantly associated in the literature with respective primary sensory and motor cortices. Voxels within each of these images were averaged to obtain 4 BOLD time series from each resting state sequence for each of the 475 subjects from the Human Connectome Project S500 release. Functional connectivity was then estimated as above for each of the four sensory and motor cortex seeds and each of the 6,923 ROIs described above. This was correlated with sleep duration for each subject as above (Fig. 2), using *q* < .05, false discovery rate for multiple comparison correction.

For all four sensory cortices, significant associations between functional connectivity and sleep duration were clustered within the basal ganglia, cerebellum, and sensory/motor cortex. To further evaluate these relationships, fourteen subject‐specific subcortical regions were defined using Freesurfer‐derived segmentation (Fischl et al., 2002) of bilateral thalamus, caudate, putamen, amygdala, hippocampus, pallidum, and nucleus accumbens. 14 cerebellar regions were added using the parcellation of Buckner et al. (Buckner, Krienen, Castellanos, Diaz, & Yeo, 2011) and using a 7‐network parcellation split into left‐ and right‐hemispheric regions, dividing the cerebellum into regions most correlated with canonical brain networks. This resulted in a total of 32 ROIs: four bilateral sensory/motor cortex, 14 unilateral subcortical, and 14 unilateral cerebellar regions. Functional connectivity was calculated for each subject between each pair of ROIs, and correlated with sleep duration as above.

To evaluate whether relationships observed in functional connectivity may be consequences of residual head motion not accounted for by FIX ICA postprocessing, we obtained head motion estimates supplied with the Minimally Preprocessed Data release for all 839 subjects. Detrended head motion time series for six realignment parameters and their first derivatives were subtracted via linear regression using a general linear model (glmfit.m in Matlab) from FIX ICA‐processed BOLD time series data for all 32 ROIs listed above in all four sequences for each of the 839 subjects in the HCP 900 Subject Release. These data were then further processed by removal of all time points before and after any volumes where greater than 0.2 mm head motion was observed in any of the six realignment parameters. Remaining motion‐regressed BOLD time series data were then concatenated to obtain motion‐corrected estimates of functional connectivity between all ROIs (Power, Barnes, Snyder, Schlaggar, & Petersen, 2012). The more rigorously processed estimates of correlation between sleep duration and functional connectivity were virtually indistinguishable from those processed with the FIX ICA pipeline alone (*r *= .9997, *p *= 1.7 e‐196).

Subjects were then grouped based on whether they reported short (≤6 hr) versus conventional (7–9 hr) sleep duration, and whether they reported daytime dysfunction. These data were derived from self‐report answers to the Pittsburgh Sleep Quality Index (PSQI), a 24‐item questionnaire comprising seven component scores including sleep duration (component 3) and daytime dysfunction (component 7) (Buysse et al., 1989). Sleep duration was obtained from question #4 of the PSQI: “During the past month, how many hours of *actual sleep* did you get at night? (This may be different than the number of hours you spend in bed.)” (p. 209 Buysse et al., 1989). Individuals denying daytime dysfunction reported scores of zero on Component 7: Daytime Dysfunction of the PSQI. This corresponds to answering “Not during the past month” to PSQI question #8: “During the past month, how often have you had trouble staying awake while driving, eating meals, or engaging in social activity?” and answering “No problem at all” to PSQI question #9: “During the past month, how much of a problem has it been for you to keep up enough enthusiasm to get things done?” (p. 210, Buysse et al., 1989). Individuals reporting daytime dysfunction reported Component 7: Daytime Dysfunction scores greater than zero. This strategy resulted in the following groups: short sleepers denying daytime dysfunction (*n *=* *115); short sleepers reporting daytime dysfunction (*n *=* *176); conventional sleepers denying daytime dysfunction (*n *=* *318); and conventional sleepers reporting daytime dysfunction (*n *=* *249). The same 32 ROIs were used to evaluate relationships between functional connectivity and group differences between short and conventional sleepers, and between subjects reporting or denying daytime dysfunction. Two‐tailed t‐tests were used with false discovery rate correction for multiple comparisons to evaluate intergroup differences between short and conventional sleepers, both reporting and denying daytime dysfunction, for each pair of the 32 ROIs.

### 2.3 Principal component analysis

Because a relatively small portion of the variance of functional connectivity was accounted for by sleep duration (correlation values ≤0.2), we performed additional analyses to determine what the primary patterns of variance were across subjects in functional connectivity, and whether changes in connectivity associated with sleep duration represented a significant variation pattern.

Functional connectivity metrics for each subject for the four primary sensory/motor seeds compared to the 32 ROIs above were linearized for each subject to represent 118 distinct ROI pairs. This resulted in a matrix of 839 subjects × 118 connectivity measurements. Singular value decomposition was used to identify principal components. These components are ranked in order of variance accounted for among individuals in functional connectivity for this set of regions. The second principal component showed striking similarity to differences in functional connectivity associated with sleep duration (*r *= .85, *p *= 1.1 * 10^−33^).

## 3 Results

We used a whole‐connectome discovery approach to study the effects of average sleep duration on brain functional connectivity by evaluating the Human Connectome Project dataset. This dataset includes resting state BOLD images for 839 subjects with complete fMRI and behavioral data, comprising four 15‐minute BOLD acquisitions and extensive subject‐specific behavioral and neuropsychological metrics. Specifically, we evaluated the association of functional connectivity with self‐reported average sleep duration obtained from items on the Pittsburgh Sleep Quality Index (Buysse et al., 1989).

Sleep duration was correlated with functional connectivity obtained from a dense parcellation of 6,923 brain regions covering cortical, subcortical, and cerebellar gray matter at a spatial resolution of 5 mm in 475 subjects (S500 Release). This resulted in 23,960,503 “connections” between brain regions. Using an acceptable false discovery rate of *q *< .05, 65,963 connections demonstrated significant correlation with sleep duration across subjects. These connections were tabulated for each ROI and are displayed in Fig. 1. Brain regions showing the most frequent involvement in connections modulated by sleep duration are primarily in the sensory and motor cortices, including auditory, visual, and sensorimotor cortices.

In order to further evaluate the association of sleep duration with functional connectivity involving sensory and motor cortices, seed locations were obtained using the NeuroSynth database, allowing identification of consensus regions associated with sensory and motor cortex in the neuroimaging literature. Four seeds were obtained, one each for primary auditory, primary motor, primary somatosensory, and primary visual cortices. Each seed included voxels from bilateral sensory or motor cortex. Connectivity was measured between each seed and the 6,923 ROIs covering brain gray matter, displayed in Fig. 2.

**Figure 3:**
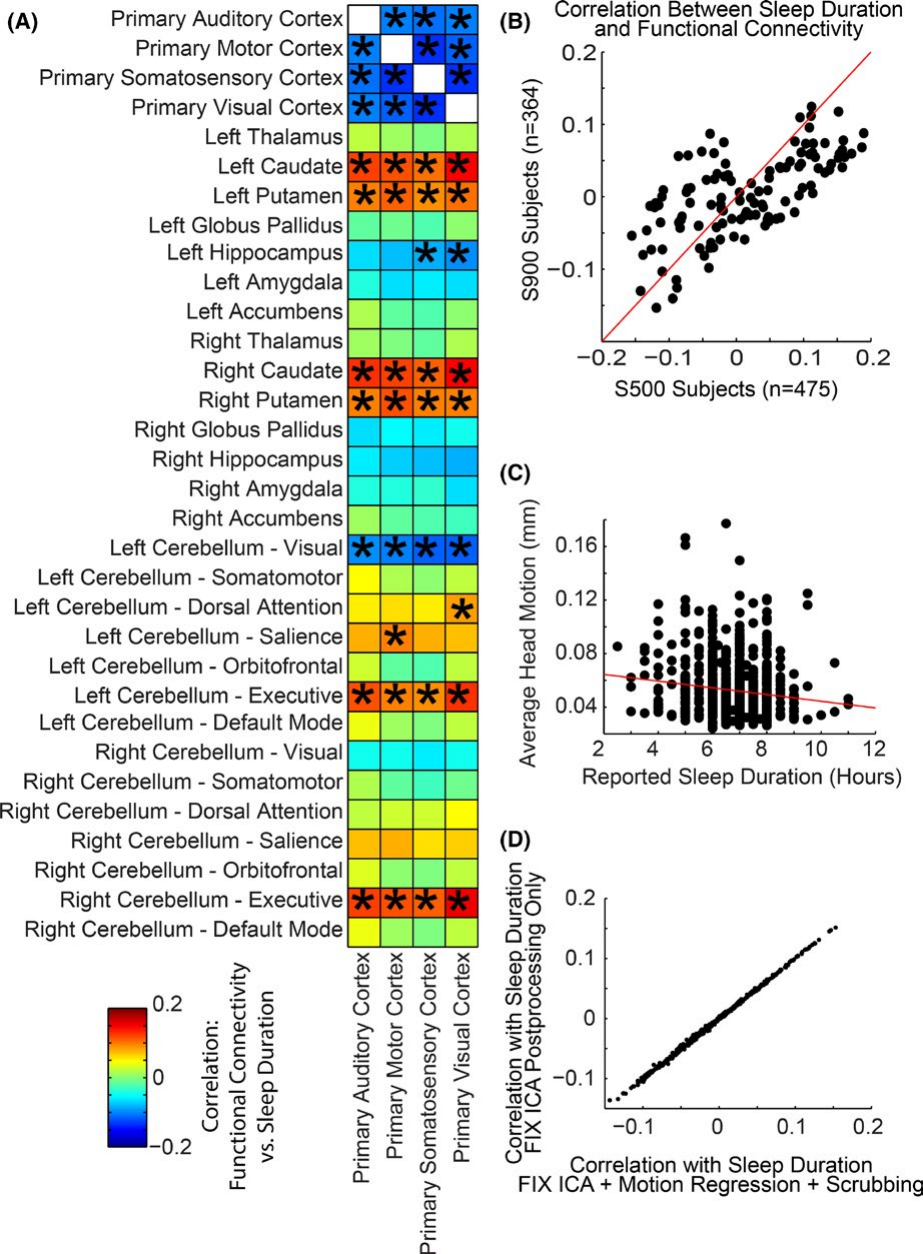
Correlation of sleep duration and functional connectivity between sensory/motor cortex, subcortical, and cerebellar regions. (A) Asterisk (*) denotes connections that were significant for false discovery rate *q* < .05 over all region pairs. Color scale shows Pearson correlation coefficient between functional connectivity and sleep duration across subjects. (B) Correlation values were obtained from two subject cohorts: *n *= 475 subjects from the S500 Release, and *n *= 364 additional subjects from the S900 Release. Scatter plots show comparison of results for the same 4 × 32 ROIs for both subject samples. (C) Relationship between sleep duration and head motion for all 839 subjects is shown. (D) Comparison of correlation with sleep duration for the same 4 × 32 ROIs using two different postprocessing strategies to account for head motion

For all four seed regions, there was a very similar distribution of brain regions that showed positive and negative correlations with sleep duration. Negative correlations were noted between sleep duration and connectivity within the remaining sensory and motor cortex, and positive correlations were noted with the cerebellum and basal ganglia. This pattern was further evaluated by extracting regions of interest representing subject‐specific gray matter ROIs for subcortical regions. Seven bilateral regions were identified for each subject from Freesurfer parcellation of structural brain imaging (Fischl et al., 2002) and BOLD time series were extracted for each region: thalamus, caudate, putamen, globus pallidus, accumbens, hippocampus, and amygdala. To evaluate the distribution of connectivity within the cerebellum, a functional parcellation of the cerebellum was used to subdivide cerebellar regions most associated with seven canonical functional brain networks (Buckner et al., 2011). In addition to the 4 sensory/motor cortex seeds previously described, this resulted in 32 regions, and functional connectivity was compared to sleep duration for connections between the four sensory/motor seeds and these 32 ROIs, shown in Fig. 3.

Consistent with whole‐brain images in Fig. 2, shorter sleep duration was associated with lower connectivity of sensory and motor cortex to the caudate, putamen, and portions of the cerebellum most connected to salience and executive networks. In contrast, higher connectivity with shorter sleep duration was seen between sensory and motor cortex and the hippocampus and other sensory/motor cortical regions.

We observed that head motion was significantly correlated with average reported sleep duration, which could potentially confound the relationship between functional connectivity and sleep duration. To investigate this possibility, we additionally processed each BOLD time series by regressing six head motion parameters and their first derivatives from the data, followed by volume censoring (scrubbing) in which time points with head motion exceeding 0.2 mm were removed and remaining data concatenated (Power et al., 2012).

Given the high data quality and large sample size of the Human Connectome Project dataset, it is possible that very subtle differences in connectivity are found in relation to sleep duration that may be of limited impact in describing functional connectivity variation among subjects. For example, all the correlations between sleep duration and functional connectivity are less than about 0.2.

**Figure 4:**
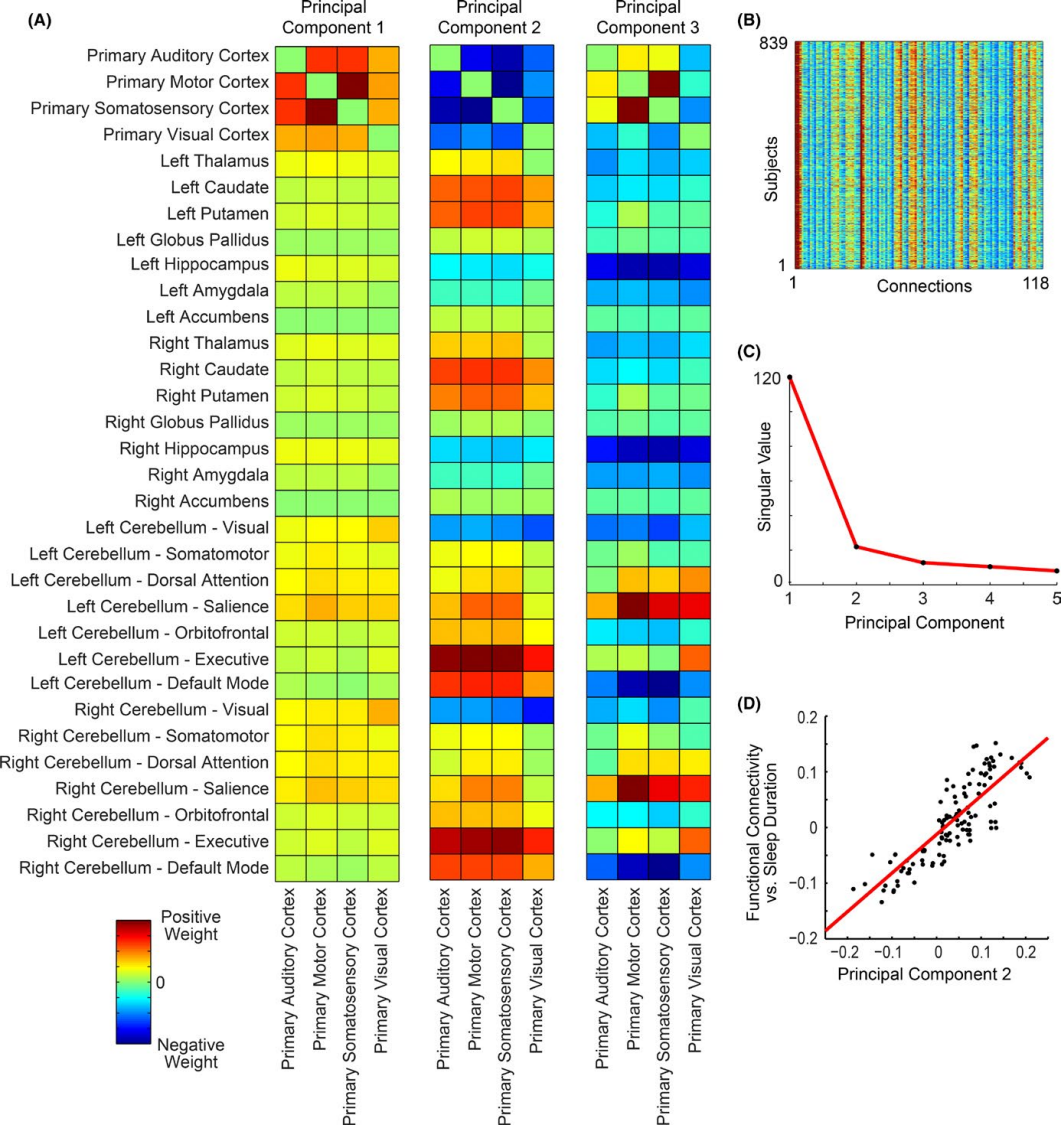
Principal components of intersubject variance in functional connectivity. (A) The first three principal components are shown representing subject by connection singular vectors. (B) Matrix of 839 subjects × 118 connections used to obtain principal components. (C) Singular values of first five components. (D) Comparison of principal component 2 with relationship of sleep duration and functional connectivity for the same connections

To quantitatively compare changes in functional connectivity with primary patterns of variation in functional connectivity in the Human Connectome Project dataset, we linearized functional connectivity measurements for each subject from the 4 × 32 ROI set described above to obtain a matrix of 839 subjects by 118 distinct connections (Fig. 4, top right). We performed principal component analysis on this matrix by performing a singular value decomposition to obtain vectors of 118 connections that represent patterns of covariation in functional connectivity contributing to the greatest percentage of intersubject variance.

The first three principal components are shown in Fig. 4, left, with singular values representing the relative contribution to explaining variance in connectivity for these ROIs between subjects (Fig. 4, right middle). The first principal component represents a mode of variation in which some subjects had relatively lower cortico‐cortical connectivity compared to cortical‐subcortical or cortical‐cerebellar connectivity. The second principal component showed a pattern strikingly similar to Fig. 3. This is quantified in Fig. 4, bottom right, in which comparison of functional connectivity to sleep duration is closely matched to the second principal component. Thus, the pattern of functional connectivity associated with sleep duration represents one of the primary patterns observed in resting connectivity within these ROIs.

**Figure 5:**
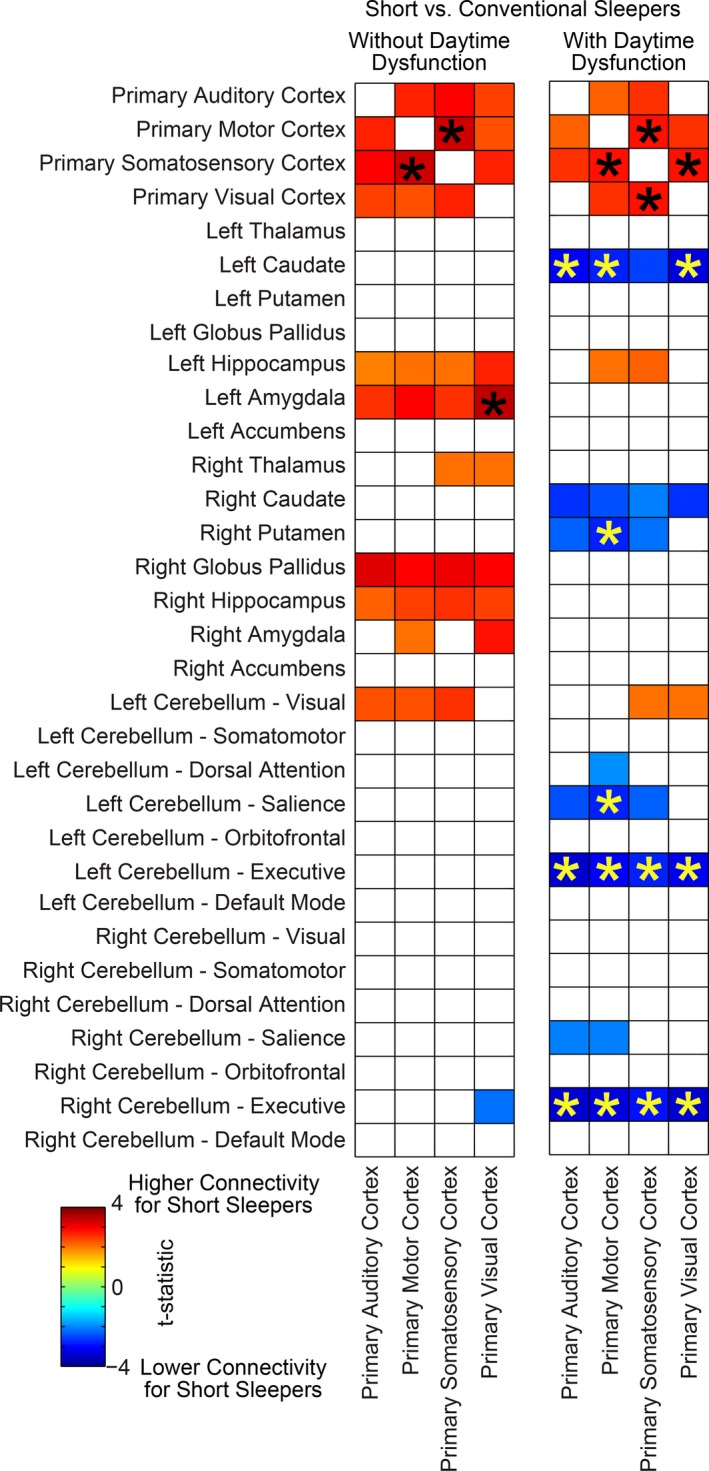
Differences in functional connectivity in self‐reported short sleepers versus conventional sleepers reporting and denying daytime dysfunction. Colored squares satisfied *p* < .05, uncorrected. Asterisk (*) denotes connections that were significant for false discovery rate *q* < .05 over all region pairs. Color scale represents t‐statistic for a two‐tailed t‐test of functional connectivity between short and conventional sleepers

To examine functional connectivity differences between self‐reported short sleepers and conventional sleepers, we divided the sample into two groups: those reporting average sleep durations ≤6 hr each night (short sleepers) and those reporting average sleep durations between 7 and 9 hr each night (conventional sleepers) – consistent with current NSF sleep duration recommendations (Hirshkowitz et al., 2015). We further divided groups into individuals reporting versus denying daytime dysfunction based on the items comprising Pittsburgh Sleep Quality Index Component 7: Daytime Dysfunction scores (Buysse et al., 1989). Results of two‐sample t‐tests to examine differences between self‐reported short versus conventional sleepers reporting and denying daytime dysfunction, is shown in Fig. 5.

A dissociation was evident between self‐reported short sleepers denying daytime dysfunction, in which modulation of sensory/motor connectivity consisted of increased connection to bilateral hippocampus and amygdala, and self‐reported short sleepers reporting daytime dysfunction, in which modulation of sensory/motor connectivity consisted of decreased connectivity to the dorsal striatum and cerebellum.

**Figure 6:**
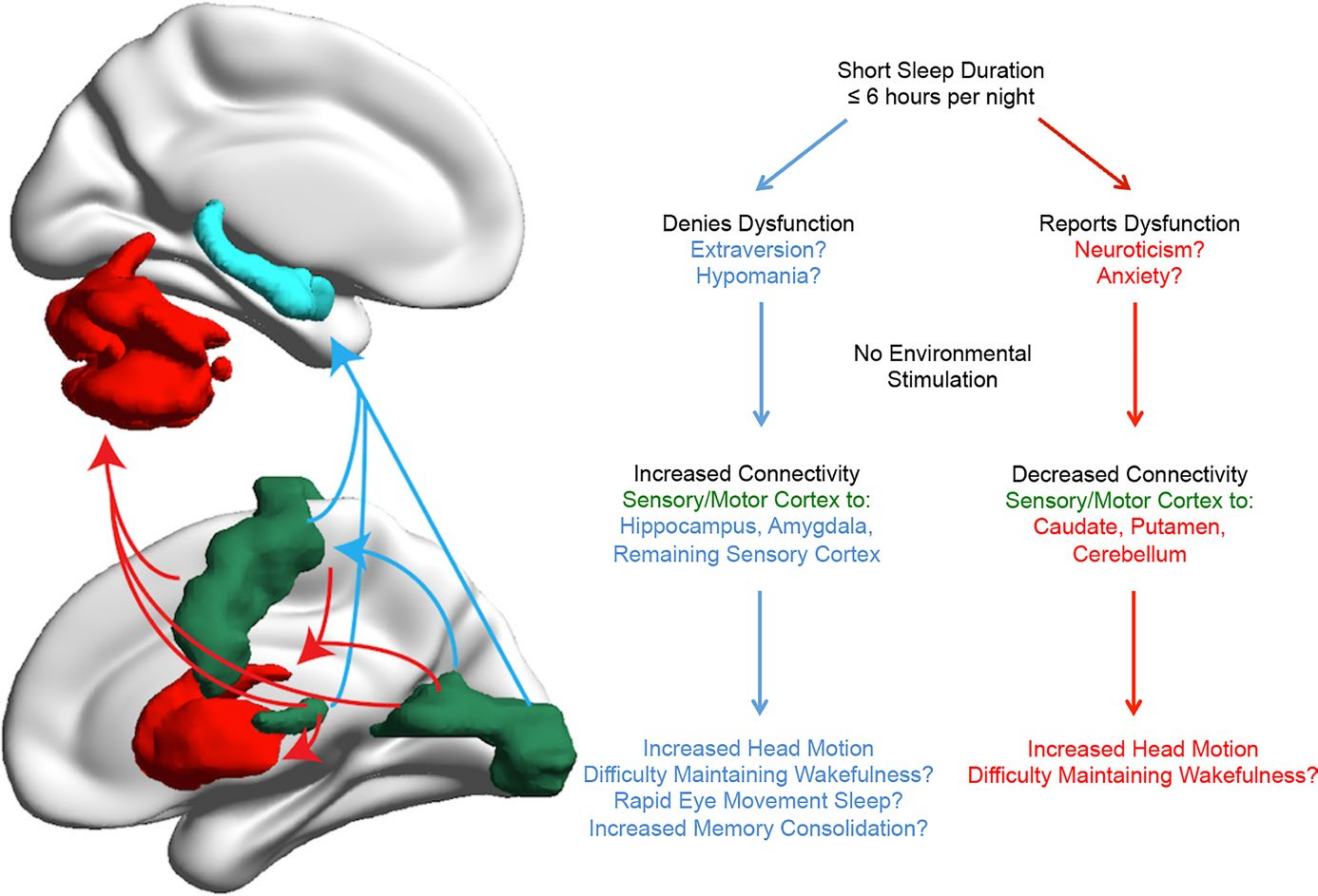
Model for effects of sleep duration on functional connectivity in self‐reported short sleepers reporting and denying daytime dysfunction

Taken together, these findings suggest that functional connectivity covaries with average sleep duration, with greatest effects in sensory and motor cortex (Figs 1, 2, 3, 4). Regions of sensory and motor cortex show differential modulation of connectivity in self‐reported short sleepers denying daytime dysfunction, exhibiting increased sensory/motor connectivity to bilateral hippocampus, amygdala, and other regions of sensory and motor cortex, and self‐reported short sleepers reporting daytime dysfunction, exhibiting decreased sensory/motor connectivity to dorsal striatum and executive cerebellum (Fig. 5). These relationships and hypothesized emotional and behavioral correlates warranting further study are summarized in Fig. 6.

## 4 Discussion

In this study, the extent to which self‐reported sleep duration is associated with brain functional connectivity differences during R‐fMRI was examined using the Human Connectome Project database (Van Essen et al., 2013). Self‐reported sleep duration primarily covaried with functional connectivity in auditory, visual, and sensorimotor cortices (Fig. 1). Shorter sleep durations were associated with increased connectivity between primary auditory, primary motor, primary somatosensory, and primary visual cortex seed regions to the hippocampus and additional sensory/motor cortical regions, with decreased connectivity to the caudate, putamen, and cerebellar salience and executive networks (Figs 2, 3). These patterns were strikingly similar to the second principal component of intersubject variance (Fig. 4D), suggesting clinicians and researchers using R‐fMRI should ask patients and research participants how many hours of actual sleep they have routinely gotten during the past month. Responses to this single question predict one of the primary patterns of brain functional connectivity differences between individuals during R‐fMRI.

Next, functional connectivity differences between self‐reported short sleepers and conventional sleepers who report versus deny daytime dysfunction (i.e., have trouble staying awake while driving, eating meals, or engaging in social activities and who also deny problems keeping up enthusiasm to get things done during the day) were examined. Both subtypes of short sleepers were found to have increased connectivity between primary auditory, primary motor, primary somatosensory, and primary visual cortices during R‐fMRI (Fig. 5). These patterns of functional connectivity suggest both subtypes of self‐reported short sleepers may have had difficulty maintaining wakefulness during R‐fMRI. The association between these functional connectivity patterns and increased head motion may be a result of attempts to maintain wakefulness. These findings are consistent with prior research examining wake‐sleep transition during R‐fMRI. For example, using combined EEG and R‐fMRI, Tagliazucchi and Laufs found one third of 71 participants fell asleep within four minutes of R‐fMRI, with increased BOLD signals in motor and sensory cortices during the transition from wakefulness to stage 1 and stage 2 sleep (Tagliazucchi & Laufs, 2014). This neural signature was previously described by Horovitz and colleagues using combined EEG and R‐fMRI (Horovitz et al., 2008), with increased BOLD signals observed primarily in visual, motor, and primary auditory cortices during the transition from resting wakefulness to sleep onset and is reproducible across laboratories (Davis et al., 2016; Larson‐Prior et al., 2009; Tagliazucchi & Laufs, 2014).

Importantly, these findings support the supposition that self‐reported short sleepers who deny daytime dysfunction may require environmental stimulation to maintain wakefulness. This is consistent with Hegerl and Hensch's model suggesting that seeking environmental stimulation is a behavioral strategy to overcome the need to sleep in individuals prone to psychopathology characterized by high behavioral activation (e.g., mania, ADHD; Hegerl & Hensch, 2014). Indeed, prior research suggests that short sleepers who deny daytime dysfunction exhibit patterns of behavioral activation levels and increased environmental stimulation seeking that may approach hypomania (Curtis et al., 2011; Hartmann et al., 1972; He et al., 2009; Jones & Oswald, 1968; Monk et al., 2001). To our knowledge, the findings of this study represent the first R‐fMRI data suggesting self‐reported short sleepers who deny daytime dysfunction may have difficulty maintaining wakefulness under conditions of low environmental stimulation. Future studies using combined EEG and R‐fMRI are warranted to definitively distinguish resting wakefulness from sleep onset in short sleeper subtypes.

Current findings are notable considering that the R‐fMRI protocol used in the Human Connectome Project database provided explicit instruction to maintain eyes open with visual fixation. Based on their combined EEG and R‐fMRI findings, Tagliazucchi and Laufs (Tagliazucchi & Laufs, 2014) developed a support vector machine classifier to determine how many subjects fell asleep during R‐fMRI using the 1000 Functional Connectomes Project database (Biswal et al., 2010) – finding approximately one third of 1,147 subjects fell asleep within 3 minutes. As noted by Tagliazucchi and Laufs, the 35 international collaborating centers comprising the 1000 Functional Connectomes Project database (Biswal et al., 2010) use one of three R‐fMRI protocols: eyes closed, eyes open, or eyes open with fixation. Centers adopting R‐fMRI protocols with eyes closed or eyes open had relatively high probabilities of falling asleep with increased scanning time, whereas the five centers adopting a R‐fMRI protocol with eyes open and fixation had extremely high probabilities of maintaining wakefulness (Fig. 4C, Tagliazucchi & Laufs, 2014). In contrast to the 1000 Functional Connectomes Project, all data from the 900 WU‐Minn Human Connectome Project database reported in this study use a single R‐fMRI protocol: eyes open with fixation and explicit instructions not to fall asleep (Smith et al., 2013). Therefore, current findings suggest high levels of sleep pressure in both self‐reported short sleepers who report daytime difficulties and those denying such difficulties, resulting in a possible loss of wakefulness under R‐fMRI conditions expected to facilitate the maintenance of a relaxed, but alert state (Aeschbach et al., 2001). Future studies applying Tagliazucchi and Laufs support vector machine classifier (Tagliazucchi et al., 2012) may be useful in determining difficulty maintaining wakefulness in short sleeper subtypes during R‐fMRI when combined EEG is not available.

Notably, current findings suggest individual differences in the accuracy of self‐reported daytime functioning. Specifically, those denying daytime sleepiness appear to have difficulty maintaining wakefulness during R‐fMRI conditions at levels comparable to short sleepers reporting daytime sleepiness. It can be hypothesized that short sleepers denying daytime dysfunction underestimate their true level of daytime sleepiness. As chronic sleep deprivation progresses, individuals tend to underestimate their subjective level of daytime impairment (Van Dongen, Maislin, Mullington, & Dinges, 2003). Chronic sleep restriction over 21 days results in linear decreases in reaction time that mirror our internal circadian rhythms (Cohen et al., 2010). Despite chronic sleep loss, reaction times reach relatively normal levels in the late afternoon, possibly leading some chronically sleep‐deprived individuals to subjectively underestimate their objective levels of impairment (Cohen et al., 2010). A similar process may be evident in self‐reported short sleepers who deny daytime dysfunction, who may seek environmental stimulation as a behavioral strategy to overcome their true level of daytime sleepiness.

Although both subgroups of short sleepers may be at risk of diminished wakefulness under conditions of low environmental stimulation, the neural mechanisms underlying group differences in subjective perceptions of daytime impairment have not been examined. Toward this end, present findings are notable in demonstrating increased connectivity between sensory cortices and bilateral hippocampus and amygdala in short sleepers denying daytime dysfunction, but not in short sleepers reporting daytime dysfunction (Fig. 5). Transient coactivation of amygdala, hippocampus, and sensory cortices are hypothesized to form an emotional‐perceptual‐memory circuit associated with instances of phasic rapid eye movement periods in humans (Wehrle et al., 2007). One of the primary hypotheses for why animals need sleep is for memory consolidation (Stickgold, 2005). Thus, increased connectivity between sensory cortex and medial temporal regions critical for memory retrieval and storage may represent a marker of memory consolidation in short sleepers denying daytime dysfunction. Interestingly, increased functional connectivity between the hippocampus and lateral occipital complex during resting wakefulness following an object‐face encoding task with high associative memory may predict individual differences in better long‐term memory performance and increased memory consolidation (Tambini, Ketz, & Davachi, 2010). Although increased neocortico‐hippocampal connectivity during R‐fMRI predicting subsequent memory retrieval is an emerging area of research (reviewed in Picchioni, Duyn, & Horovitz, 2013), an increased ability for memory consolidation during evening sleep or multiple daytime “microsleeps” (Hegerl & Hensch, 2014; Van Sweden, 1986) may partially explain perceptions of functionality despite short total sleep time in this subgroup. Although definitive sleep onset and memory consolidation cannot be determined by present findings, these results suggest short sleepers denying daytime dysfunction may have more efficient homeostatic sleep regulation than short sleepers reporting dysfunction (e.g., He et al., 2009), enabling them to subjectively feel alert during the day despite short sleep duration. These hypotheses warrant further study.

The majority of neuroimaging studies to date have examined functional connectivity differences following partial sleep restriction or total sleep deprivation under laboratory‐controlled conditions. As others have noted, there is a need for more ecologically valid research on the effects of chronic partial sleep deprivation in “real world” settings (Grandner, Patel, et al., 2010). Consistent with the current findings, Kilgore and colleagues conducted one of the few neuroimaging studies examining functional connectivity differences in subjects adhering to their typical sleep schedules and found shorter self‐reported sleep duration the night before a R‐fMRI scan was associated with greater connectivity between the posterior cingulate cortex and bilateral parietal lobe, middle occipital gyrus, and inferior postcentral sulcus (Killgore, Schwab, & Weiner, 2012). Killgore and colleagues selected seed regions in the medial prefrontal cortex and posterior cingulate cortex *a priori*, based on regions of the default mode network previously implicated in experimental sleep deprivation. It remains unknown whether their findings would validate or contradict present findings if seed regions were placed in primary auditory, primary motor, primary somatosensory, and primary visual cortices (Fig. 5).

The findings of this study should not be generalized beyond self‐reports of sleep duration. Future studies should examine “verified short sleepers” using objective methods such as actigraphy or polysomnography to quantify sleep parameters on workdays and nonworkdays (Grandner, Patel, et al., 2010). We agree with these recommendations, and have reported data on actigraphy‐verified short sleepers (Curtis et al., 2011; He et al., 2009). However, the Human Connectome Project database offers the opportunity to examine brain functional connectivity differences in relation to sleep duration in a large sample. Although actigraphy and sleep diary data are not available in this study, PSQI sleep duration scores have been shown to correlate significantly (Spearman's *rho* = −0.204; *p *<* *.05) with actigraphic total sleep times in adults not selected for sleep disturbance (Grandner, Kripke, Yoon, & Youngstedt, 2006) – selection criteria mirroring the Human Connectome Project database (Van Essen et al., 2013). Similarly, PSQI daytime dysfunction scores have been shown to correlate significantly with the Epworth Sleepiness Scale (ESS; correlation coefficient = 0.34; *p *<* *.001; Buysse et al., 2008), with the ESS (Johns, 1991) being one of the gold standard instruments used to determine excessive daytime sleepiness in humans (Johns, 2000). A final limitation of this study involves inferring wakefulness using R‐fMRI without simultaneous EEG recordings. As noted previously, however, BOLD signals in sensory areas of the brain including visual, motor, and primary auditory cortices can be used as “a surrogate indicator of wakefulness when EEG is not available” (p. 680 Horovitz et al., 2008). Future studies using combined EEG and R‐fMRI or applying Tagliazucchi and Laufs support vector machine classifier to existing R‐fMRI data (Tagliazucchi et al., 2012) are warranted to further explore difficulty maintaining wakefulness without environmental stimulation in short sleeper subtypes.

In conclusion, our findings indicate that even in a healthy population, self‐reported sleep duration predicts one of the primary patterns of brain functional connectivity observed during R‐fMRI (Fig. 4). This finding has widespread implications for the interpretation and study design of fMRI investigations, indicating researchers using R‐fMRI should routinely ask how many hours of sleep per night participants have gotten during the past month and consider objective measurements of whether participants may be falling asleep in the scanner.

Self‐report surveys suggest 30% of working adults in the United States routinely get 6 hours or less sleep, on average, in a 24‐hour period (Luckhaupt et al., 2010). Similarly, 291 of 970 subjects (30%) in the Human Connectome Project database reported sleeping 6 hr or less each night, on average, during the past month. Findings from this study suggest that epidemiological research on self‐reported sleep duration should differentiate those who report versus deny perceived daytime dysfunction. Although this study suggests short sleepers may have difficulty maintaining an alert state during resting assessment, future fMRI research should examine actigraphy‐verified short sleeper subtypes and conventional sleepers using coincident EEG recordings to validate and extend the present findings.

To the extent that current findings can be generalized to other low environmental stimulation conditions, such as driving an automobile at night without sufficient visual or auditory stimulation, the public health implications of these findings are clear. Regardless of whether individuals perceive sleep‐related daytime difficulties, short sleep duration may confer increased risk of drowsiness in situations characterized by low environmental stimulation. Furthermore, our findings demonstrate R‐fMRI differences between short sleeper subtypes, offering preliminary insight into the neural mechanisms underlying differences in subjective perceptions of daytime impairment resulting from short sleep duration.

## Funding Information

National Institute of Mental Health, (Grant / Award Number: ‘K08092697′, ’R01 MH080826’).

## Conflicts of Interest

None declared.

## References

[brb3576-bib-0001] Aeschbach, D. , Postolache, T. T. , Sher, L. , Matthews, J. R. , Jackson, M. A. , & Wehr, T. A. (2001). Evidence from the waking electroencephalogram that short sleepers live under higher homeostatic sleep pressure than long sleepers. Neuroscience, 102, 493–502. 11226688 10.1016/s0306-4522(00)00518-2

[brb3576-bib-0002] American Academy of Sleep Medicine ed. (2014). International classification of sleep disorders, 3rd ed Darien, IL: American Academy of Sleep Medicine.

[brb3576-bib-0003] Biswal, B. B. , Mennes, M. , Zuo, X.‐N. , Gohel, S. , Kelly, C. , Smith, S. M. , … Milham, M. P. (2010). Toward discovery science of human brain function. Proceedings of the National Academy of Sciences of the United States of America, 107, 4734–4739. 20176931 10.1073/pnas.0911855107 PMC2842060

[brb3576-bib-0004] Buckner, R. L. , Krienen, F. M. , Castellanos, A. , Diaz, J. C. , & Yeo, B. T. T. (2011). The organization of the human cerebellum estimated by intrinsic functional connectivity. Journal of Neurophysiology, 106, 2322–2345. 21795627 10.1152/jn.00339.2011 PMC3214121

[brb3576-bib-0005] Buysse, D. J. , Hall, M. L. , Strollo, P. J. , Kamarck, T. W. , Owens, J. , Lee, L. , … Matthews, K. A. (2008). Relationships between the Pittsburgh Sleep Quality Index (PSQI), Epworth Sleepiness Scale (ESS), and clinical/polysomnographic measures in a community sample. Journal of Clinical Sleep Medicine, 4, 563–571. 19110886 PMC2603534

[brb3576-bib-0006] Buysse, D. J. , Reynolds, C. F. , Monk, T. H. , Berman, S. R. , & Kupfer, D. J. (1989). The Pittsburgh Sleep Quality Index: A new instrument for psychiatric practice and research. Psychiatry Research, 28, 193–213. 2748771 10.1016/0165-1781(89)90047-4

[brb3576-bib-0007] Cohen, D. A. , Wang, W. , Wyatt, J. K. , Kronauer, R. E. , Dijk, D.‐J. , Czeisler, C. A. , & Klerman, E. B. (2010). Uncovering residual effects of chronic sleep loss on human performance. Science Translational Medicine, 2, 14ra3–14ra3. 10.1126/scitranslmed.3000458 PMC2892834 20371466

[brb3576-bib-0008] Curtis, B. J. , Brewer, J. A. , & Jones, C. R. (2011). Short sleeper syndrome (SSS): A possible sleep‐duration, circadian, metabolic, affective, pain‐tolerance, normal variant in humans. Sleep, 34, A259–A260.

[brb3576-bib-0009] Davis, B. , Tagliazucchi, E. , Jovicich, J. , Laufs, H. , & Hasson, U. (2016). Progression to deep sleep is characterized by changes to BOLD dynamics in sensory cortices. NeuroImage, 130, 293–305. 26724779 10.1016/j.neuroimage.2015.12.034 PMC4819724

[brb3576-bib-0010] Dorsey, C. M. , & Bootzin, R. R. (1997). Subjective and psychophysiologic insomnia: An examination of sleep tendency and personality. Biological Psychiatry, 41, 209–216. 9018392 10.1016/0006-3223(95)00659-1

[brb3576-bib-0011] Duggan, K. A. , Friedman, H. S. , McDevitt, E. A. , & Mednick, S. C. (2014). Personality and healthy sleep: The importance of conscientiousness and neuroticism. Ed. Oscar Arias‐Carrion PLoS ONE, 9, e90628. 24651274 10.1371/journal.pone.0090628 PMC3961248

[brb3576-bib-0012] Ertel, K. A. , Berkman, L. F. , & Buxton, O. M. (2011). Socioeconomic status, occupational characteristics, and sleep duration in African/Caribbean immigrants and US White health care workers. Sleep, 34, 509–518. 21461330 10.1093/sleep/34.4.509 PMC3065262

[brb3576-bib-0013] Feinberg, D. A. , Moeller, S. , Smith, S. M. , Auerbach, E. , Ramanna, S. , Gunther, M. , … Yacoub, E. (2010). Multiplexed echo planar imaging for sub‐second whole brain FMRI and fast diffusion imaging. Ed. Pedro Antonio Valdes‐Sosa PLoS ONE, 5, e15710. 21187930 10.1371/journal.pone.0015710 PMC3004955

[brb3576-bib-0014] Fischl, B. , Salat, D. H. , Busa, E. , Albert, M. , Dieterich, M. , Haselgrove, C. , … Dale, A. M. (2002). Whole brain segmentation: Automated labeling of neuroanatomical structures in the human brain. Neuron, 33, 341–355. 11832223 10.1016/s0896-6273(02)00569-x

[brb3576-bib-0015] Glasser, M. F. , Sotiropoulos, S. N. , Wilson, J. A. , Coalson, T. S. , Fischl, B. , Andersson, J. L. , … WU‐Minn HCP Consortium (2013). The minimal preprocessing pipelines for the Human Connectome Project. NeuroImage, 80, 105–124. 23668970 10.1016/j.neuroimage.2013.04.127 PMC3720813

[brb3576-bib-0016] Grandner, M. A. , Chakravorty, S. , Perlis, M. L. , Oliver, L. , & Gurubhagavatula, I. (2014). Habitual sleep duration associated with self‐reported and objectively determined cardiometabolic risk factors. Sleep Medicine, 15, 42–50. 24333222 10.1016/j.sleep.2013.09.012 PMC3947242

[brb3576-bib-0017] Grandner, M. A. , Hale, L. , Moore, M. , & Patel, N. P. (2010). Mortality associated with short sleep duration: The evidence, the possible mechanisms, and the future. Sleep Medicine Reviews, 14, 191–203. 19932976 10.1016/j.smrv.2009.07.006 PMC2856739

[brb3576-bib-0018] Grandner, M. A. , Jackson, N. J. , Pak, V. M. , & Gehrman, P. R. (2012). Sleep disturbance is associated with cardiovascular and metabolic disorders. Journal of Sleep Research, 21, 427–433. 22151079 10.1111/j.1365-2869.2011.00990.x PMC3703752

[brb3576-bib-0019] Grandner, M. A. , Kripke, D. F. , Yoon, I.‐Y. , & Youngstedt, S. D. (2006). Criterion validity of the Pittsburgh Sleep Quality Index: Investigation in a non‐clinical sample. Sleep and Biological Rhythms, 4, 129–139. 22822303 10.1111/j.1479-8425.2006.00207.x PMC3399671

[brb3576-bib-0020] Grandner, M. A. , Patel, N. P. , Gehrman, P. R. , Perlis, M. L. , & Pack, A. I. (2010). Problems associated with short sleep: Bridging the gap between laboratory and epidemiological studies. Sleep Medicine Reviews, 14, 239–247. 19896872 10.1016/j.smrv.2009.08.001 PMC2888649

[brb3576-bib-0021] Grandner, M. A. , Sands‐Lincoln, M. R. , Pak, V. M. , & Garland, S. N. (2013). Sleep duration, cardiovascular disease, and proinflammatory biomarkers. Nature and Science of Sleep, 5, 93–107. 10.2147/NSS.S31063 PMC3724567 23901303

[brb3576-bib-0022] Griffanti, L. , Salimi‐Khorshidi, G. , Beckmann, C. F. , Auerbach, E. J. , Douaud, G. , Sexton, C. E. , … Smith, S. M. (2014). ICA‐based artefact removal and accelerated fMRI acquisition for improved resting state network imaging. NeuroImage, 95, 232–247. 24657355 10.1016/j.neuroimage.2014.03.034 PMC4154346

[brb3576-bib-0023] Hartmann, E. , Baekeland, F. , & Zwilling, G. R. (1972). Psychological differences between long and short sleepers. Archives of General Psychiatry, 26, 463–468. 5019885 10.1001/archpsyc.1972.01750230073014

[brb3576-bib-0024] He, Y. , Jones, C. R. , Fujiki, N. , Xu, Y. , Guo, B. , Holder, J. L. , … Fu, Y.‐H. (2009). The transcriptional repressor DEC2 regulates sleep length in mammals. Science, 325, 866–870. 19679812 10.1126/science.1174443 PMC2884988

[brb3576-bib-0025] Hegerl, U. , & Hensch, T. (2014). The vigilance regulation model of affective disorders and ADHD. Neuroscience and Biobehavioral Reviews, 44, 45–57. 23092655 10.1016/j.neubiorev.2012.10.008

[brb3576-bib-0026] Hirshkowitz, M. , Whiton, K. , Albert, S. M. , Alessi, C. , & Bruni, O. (2015). National Sleep Foundation's sleep time duration recommendations: Methodology and results summary. Sleep Health, 1, 40–43. 10.1016/j.sleh.2014.12.010 29073412

[brb3576-bib-0027] Horovitz, S. G. , Fukunaga, M. , de Zwart, J. A. , van Gelderen, P. , Fulton, S. C. , Balkin, T. J. , & Duyn, J. H. (2008). Low frequency BOLD fluctuations during resting wakefulness and light sleep: A simultaneous EEG‐fMRI study. Human Brain Mapping, 29, 671–682. 17598166 10.1002/hbm.20428 PMC6871022

[brb3576-bib-0028] Jesmanowicz, A. , Nencka, A. S. , Li, S.‐J. , & Hyde, J. S. (2011). Two‐axis acceleration of functional connectivity magnetic resonance imaging by parallel excitation of phase‐tagged slices and Half k‐Space Acceleration. Brain Connectivity, 1, 81–90. 22432957 10.1089/brain.2011.0004 PMC3312804

[brb3576-bib-0029] Johns, M. W. (1991). A new method for measuring daytime sleepiness: The Epworth sleepiness scale. Sleep, 14, 540–545. 1798888 10.1093/sleep/14.6.540

[brb3576-bib-0030] Johns, M. W. (2000). Sensitivity and specificity of the multiple sleep latency test (MSLT), the maintenance of wakefulness test and the epworth sleepiness scale: Failure of the MSLT as a gold standard. Journal of Sleep Research, 9, 5–11. 10733683 10.1046/j.1365-2869.2000.00177.x

[brb3576-bib-0031] Jones, H. S. , & Oswald, I. (1968). Two cases of healthy insomnia. Electroencephalography and Clinical Neurophysiology, 24, 378–380. 4174010 10.1016/0013-4694(68)90199-5

[brb3576-bib-0032] Killgore, W. D. S. , Schwab, Z. J. , & Weiner, M. R. (2012). Self‐reported nocturnal sleep duration is associated with next‐day resting state functional connectivity. NeuroReport, 23, 741–745. 22872066 10.1097/WNR.0b013e3283565056

[brb3576-bib-0033] Kurien, P. A. , Chong, S. Y. C. , Ptáček, L. J. , & Fu, Y.‐H. (2013). Sick and tired: How molecular regulators of human sleep schedules and duration impact immune function. Current Opinion in Neurobiology, 23, 873–879. 23702243 10.1016/j.conb.2013.04.014 PMC3766463

[brb3576-bib-0034] Larson‐Prior, L. J. , Zempel, J. M. , Nolan, T. S. , Prior, F. W. , Snyder, A. Z. , & Raichle, M. E. (2009). Cortical network functional connectivity in the descent to sleep. Proceedings of the National Academy of Sciences of the United States of America, 106, 4489–4494. 19255447 10.1073/pnas.0900924106 PMC2657465

[brb3576-bib-0035] Lim, J. , & Dinges, D. F. (2010). A meta‐analysis of the impact of short‐term sleep deprivation on cognitive variables. Psychological Bulletin, 136, 375–389. 20438143 10.1037/a0018883 PMC3290659

[brb3576-bib-0036] Luckhaupt, S. E. , Tak, S. , & Calvert, G. M. (2010). The prevalence of short sleep duration by industry and occupation in the National Health Interview Survey. Sleep, 33, 149–159. 20175398 10.1093/sleep/33.2.149 PMC2817902

[brb3576-bib-0037] Markwald, R. R. , Melanson, E. L. , Smith, M. R. , Higgins, J. , Perreault, L. , Eckel, R. H. , & Wright, K. P. (2013). Impact of insufficient sleep on total daily energy expenditure, food intake, and weight gain. Proceedings of the National Academy of Sciences of the United States of America, 110, 5695–5700. 23479616 10.1073/pnas.1216951110 PMC3619301

[brb3576-bib-0038] Moeller, S. , Yacoub, E. , Olman, C. A. , Auerbach, E. , Strupp, J. , Harel, N. , & Ugurbil, K. (2010). Multiband multislice GE‐EPI at 7 tesla, with 16‐fold acceleration using partial parallel imaging with application to high spatial and temporal whole‐brain fMRI. Magnetic Resonance in Medicine, 63, 1144–1153. 20432285 10.1002/mrm.22361 PMC2906244

[brb3576-bib-0039] Monk, T. H. , Buysse, D. J. , Welsh, D. K. , Kennedy, K. S. , & Rose, L. R. (2001). A sleep diary and questionnaire study of naturally short sleepers. Journal of Sleep Research, 10, 173–179. 11696070 10.1046/j.1365-2869.2001.00254.x

[brb3576-bib-0040] Picchioni, D. , Duyn, J. H. , & Horovitz, S. G. (2013). Sleep and the functional connectome. NeuroImage, 80, 387–396. 23707592 10.1016/j.neuroimage.2013.05.067 PMC3733088

[brb3576-bib-0041] Power, J. D. , Barnes, K. A. , Snyder, A. Z. , Schlaggar, B. L. , & Petersen, S. E. (2012). Spurious but systematic correlations in functional connectivity MRI networks arise from subject motion. NeuroImage, 59, 2142–2154. 22019881 10.1016/j.neuroimage.2011.10.018 PMC3254728

[brb3576-bib-0042] Setsompop, K. , Gagoski, B. A. , Polimeni, J. R. , Witzel, T. , Wedeen, V. J. , & Wald, L. L. (2012). Blipped‐controlled aliasing in parallel imaging for simultaneous multislice echo planar imaging with reduced g‐factor penalty. Magnetic Resonance in Medicine, 67, 1210–1224. 21858868 10.1002/mrm.23097 PMC3323676

[brb3576-bib-0043] Shah, L. M. , Cramer, J. A. , Ferguson, M. A. , Birn, R. M. , & Anderson, J. S. (2016). Reliability and reproducibility of individual differences in functional connectivity acquired during task and resting state. Brain and Behavior, 6, e00456. 27069771 10.1002/brb3.456 PMC4814225

[brb3576-bib-0044] Smith, S. M. , Beckmann, C. F. , Andersson, J. , Auerbach, E. J. , Bijsterbosch, J. , Douaud, G. , … WU‐Minn HCP Consortium (2013). Resting‐state fMRI in the Human Connectome Project. NeuroImage, 80, 144–168. 23702415 10.1016/j.neuroimage.2013.05.039 PMC3720828

[brb3576-bib-0045] Stickgold, R. (2005). Sleep‐dependent memory consolidation. Nature, 437, 1272–1278. 16251952 10.1038/nature04286

[brb3576-bib-0046] Tagliazucchi, E. , & Laufs, H. (2014). Decoding wakefulness levels from typical fMRI resting‐state data reveals reliable drifts between wakefulness and sleep. Neuron, 82, 695–708. 24811386 10.1016/j.neuron.2014.03.020

[brb3576-bib-0047] Tagliazucchi, E. , von Wegner, F. , Morzelewski, A. , Borisov, S. , Jahnke, K. , & Laufs, H. (2012). Automatic sleep staging using fMRI functional connectivity data. NeuroImage, 63, 63–72. 22743197 10.1016/j.neuroimage.2012.06.036

[brb3576-bib-0048] Tambini, A. , Ketz, N. , & Davachi, L. (2010). Enhanced brain correlations during rest are related to memory for recent experiences. Neuron, 65, 280–290. 20152133 10.1016/j.neuron.2010.01.001 PMC3287976

[brb3576-bib-0049] Van Dongen, H. P. A. , Maislin, G. , Mullington, J. M. , & Dinges, D. F. (2003). The cumulative cost of additional wakefulness: Dose‐response effects on neurobehavioral functions and sleep physiology from chronic sleep restriction and total sleep deprivation. Sleep, 26, 117–126. 12683469 10.1093/sleep/26.2.117

[brb3576-bib-0050] Van Essen, D. C. , Smith, S. M. , Barch, D. M. , Behrens, T. E. J. , Yacoub, E. , Ugurbil, K. , & WU‐Minn HCP Consortium (2013). The WU‐Minn Human Connectome Project: An overview. NeuroImage, 80, 62–79. 23684880 10.1016/j.neuroimage.2013.05.041 PMC3724347

[brb3576-bib-0051] Van Sweden, B. (1986). Disturbed vigilance in mania. Biological Psychiatry, 21, 311–313. 3947712 10.1016/0006-3223(86)90052-1

[brb3576-bib-0052] Waters, F. , & Bucks, R. S. (2011). Neuropsychological effects of sleep loss: Implication for neuropsychologists. Journal of the International Neuropsychological Society, 17, 571–586. 21554786 10.1017/S1355617711000610

[brb3576-bib-0053] Watson, N. F. , Harden, K. P. , Buchwald, D. , Vitiello, M. V. , Pack, A. I. , Strachan, E. , & Goldberg, J. (2014). Sleep duration and depressive symptoms: A gene‐environment interaction. Sleep, 37, 351–358. 24497663 10.5665/sleep.3412 PMC3900629

[brb3576-bib-0054] Wehrle, R. , Kaufmann, C. , Wetter, T. C. , Holsboer, F. , Auer, D. P. , Pollmächer, T. , & Czisch, M. (2007). Functional microstates within human REM sleep: First evidence from fMRI of a thalamocortical network specific for phasic REM periods. European Journal of Neuroscience, 25, 863–871. 17328781 10.1111/j.1460-9568.2007.05314.x

[brb3576-bib-0055] Wulff, K. , Gatti, S. , Wettstein, J. G. , & Foster, R. G. (2010). Sleep and circadian rhythm disruption in psychiatric and neurodegenerative disease. Nature Reviews Neuroscience, 11, 589–599. 20631712 10.1038/nrn2868

[brb3576-bib-0056] Yarkoni, T. , Poldrack, R. A. , Nichols, T. E. , Van Essen, D. C. , & Wager, T. D. (2011). Large‐scale automated synthesis of human functional neuroimaging data. Nature Methods, 8, 665–670. 21706013 10.1038/nmeth.1635 PMC3146590

