# Supplementary material for: Sleep duration and resting fMRI functional connectivity: examination of short sleepers with and without perceived daytime dysfunction
Source: Brain Behav. 2016 Sep 15;6(12):e00576. doi: 10.1002/brb3.576 (PMC5166999; doi:10.1002/brb3.576)
Supplement: Supplementary file 1 [file BRB3-6-e00576-s001.docx]

**Supporting Information**

*Reproducibility of relationships between functional connectivity, sleep duration, and daytime dysfunction*

To assess the reproducibility of findings reported in this report, we performed an additional split group analysis. The 839-subject sample used for the analysis was divided randomly into 2 subgroups. The subgroups included subject totals as outlined below in Table S1.

*Table S1. Subject totals for each subgroup for split group analysis.*

| Subject Totals | Habitual Short Sleepers | | Conventional Sleepers | |
| --- | --- | --- | --- | --- |
|  | Reporting Daytime Dysfunction | Denying Daytime Dysfunction | Reporting Daytime Dysfunction | Denying Daytime Dysfunction |
| Group 1 | 75 | 52 | 119 | 96 |
| Group 2 | 73 | 48 | 98 | 114 |

Correlation between sleep duration and functional connectivity was reported for each group, using the same set of 4 x 32 ROIs displayed in Figure 3. These results are shown below in Figure S1. Connections that showed correlation with sleep duration satisfying p<0.05 in both groups included decreased correlations among primary sensory regions and left cerebellar visual regions, and increased correlations between primary sensory regions and basal ganglia and executive cerebellum.

When subgroups were considered for each group that met criteria for habitual short sleepers or conventional sleepers, decreased correlations were seen between executive cerebellum and primary sensory regions for short sleepers vs. conventional sleepers reporting daytime dysfunction, while increased connectivity between hippocampus and amygdala and primary sensory regions was primarily observed in short sleepers vs. conventional sleepers denying daytime dysfunction, shown in Figure S2.

*Factors associated with short sleep duration*

The Human Connectome Project database provides restricted access data on self-reported annual income and employment status, restricted access data on objective health measures (e.g., body mass index, blood pressure), and a non-restricted objective measure of fluid intelligence (i.e., correct responses on Penn Progressive Matrices) that may influence individual differences in sleep duration and perceived daytime dysfunction. Data on the specific occupation of participants (e.g., shift work) is not available. Multivariable regression indicates employment status and fluid intelligence predict self-reported sleep duration in the present sample, representing 0.45% and 0.76% of unique variance in sleep duration, respectively (Table S2 below). Self-reported income was the only factor found to significantly predict self-reported daytime dysfunction, representing 0.88% of unique variance (Table S3 below).

Results of comparing these factors between the subgroups of short and conventional sleepers reported in Figure 5 of our manuscript are included in Figure S3 below. These data indicate higher self-reported income in conventional sleepers denying daytime dysfunction (CS-DD) compared to habitual short sleepers denying daytime dysfunction (HSS-DD; *t*(431) = -2.33, p = .020, *d* = 0.25; Figure S3A), higher body mass index in HSS-DD compared to CS-DD (*t*(430) = 3.23, p = .001, *d* = 0.35; Figure S3C), higher systolic blood pressure in HSS-DD compared to CS-DD (*t*(423) = 2.58, p = .010, *d* = 0.27; Figure S3D), higher fluid intelligence in CS-DD compared to HSS-DD (*t*(428) = -3.74, p < .001, *d* = 0.41; Figure S3F), and higher fluid intelligence in conventional sleepers reporting daytime dysfunction (CS-RD) compared to habitual short sleepers reporting daytime dysfunction (HSS-RD; *t*(421) = -2.55, p = .011, *d* = 0.25; Figure S3F). All other comparisons were non-significant.

| Table S2. Predictors of Self-Reported Sleep Duration (Pittsburgh Sleep Quality Index) | | | | | | |
| --- | --- | --- | --- | --- | --- | --- |
| Parameter | *B* | SE *B* | 95% Lower CI | 95% Upper CI | β | % Variance |
| Intercept | 6.820*** | .038 | 6.746 | 6.894 |  |  |
| Income | .029 | .019 | -.008 | .065 | .053 | 0.25 |
| **Employment** | **-.109*** | **.054** | **-.214** | **-.004** | **-.070** | **0.45** |
| Body Mass Index | -.012 | .008 | -.027 | .003 | -.058 | 0.26 |
| BP Systolic | .002 | .004 | -.005 | .010 | .032 | 0.05 |
| BP Diastolic | -.002 | .005 | -.011 | .008 | -.017 | 0.01 |
| **Fluid Intelligence** | **.022**** | **.008** | **.006** | **.037** | **.090** | **0.76** |
| R^2^ |  |  |  |  |  | .019 |
| F |  |  |  |  |  | 3.051** |
| Note: N = 940. Multivariable regression. B = unstandardized regression coefficient. CI = confidence interval. β = standardized regression coefficient. % Variance = percent of unique variance (squared semipartial correlation) predicting self-reported sleep duration. All predictors are centered at their means.  *p < .05, **p < .01, ***p < .001 | | | | | | |

| Table S3. Predictors of Self-Reported Daytime Dysfunction (Pittsburgh Sleep Quality Index) | | | | | | |
| --- | --- | --- | --- | --- | --- | --- |
| Parameter | *B* | SE *B* | 95% Lower CI | 95% Upper CI | β | % Variance |
| Intercept | .783*** | .033 | .719 | .847 |  |  |
| **Income** | **-.047**** | **.016** | **-.079** | **-.015** | **-.102** | **0.88** |
| Employment | .043 | .046 | -.047 | .134 | .032 | 0.10 |
| Body Mass Index | .009 | .007 | -.004 | .022 | .050 | 0.20 |
| BP Systolic | -.001 | .003 | -.007 | .006 | -.009 | 0.00 |
| BP Diastolic | -.005 | .004 | -.014 | .003 | -.060 | 0.18 |
| Fluid Intelligence | .012 | .007 | -.002 | .026 | .058 | 0.32 |
| R^2^ |  |  |  |  |  | .015 |
| F |  |  |  |  |  | 2.410* |
| Note: N = 940. Multivariable regression. B = unstandardized regression coefficient. CI = confidence interval. β = standardized regression coefficient. % Variance = percent of unique variance (squared semipartial correlation) predicting self-reported daytime dysfunction. All predictors are centered at their means. Daytime Dysfunction = raw scores.  *p < .05, **p < .01, ***p < .001 | | | | | | |


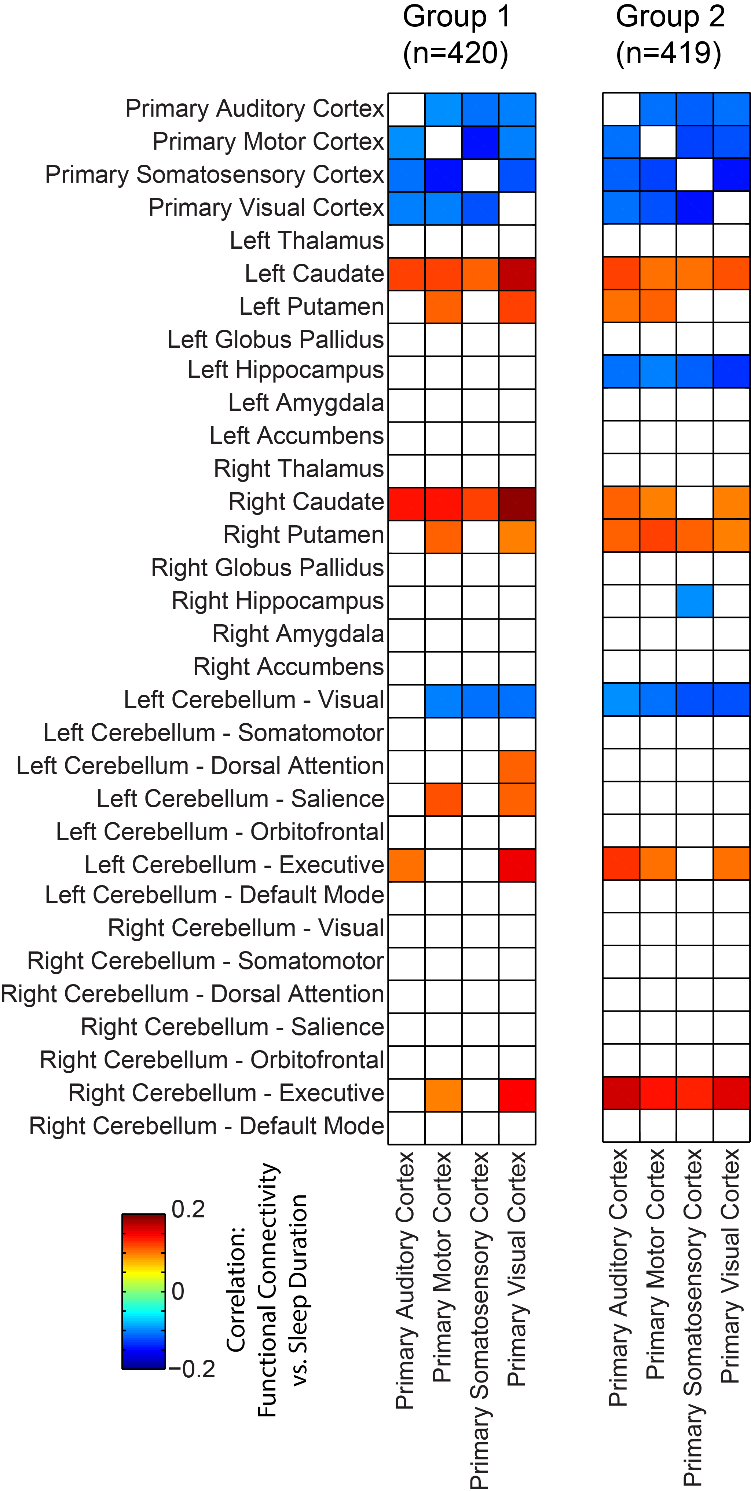


Figure S1: Correlation of sleep duration and functional connectivity between sensory/motor cortex, subcortical, and cerebellar regions for 2 subgroups of subjects. Color scale shows Pearson correlation coefficient between functional connectivity and sleep duration across subjects, thresholded at p<0.05, uncorrected.


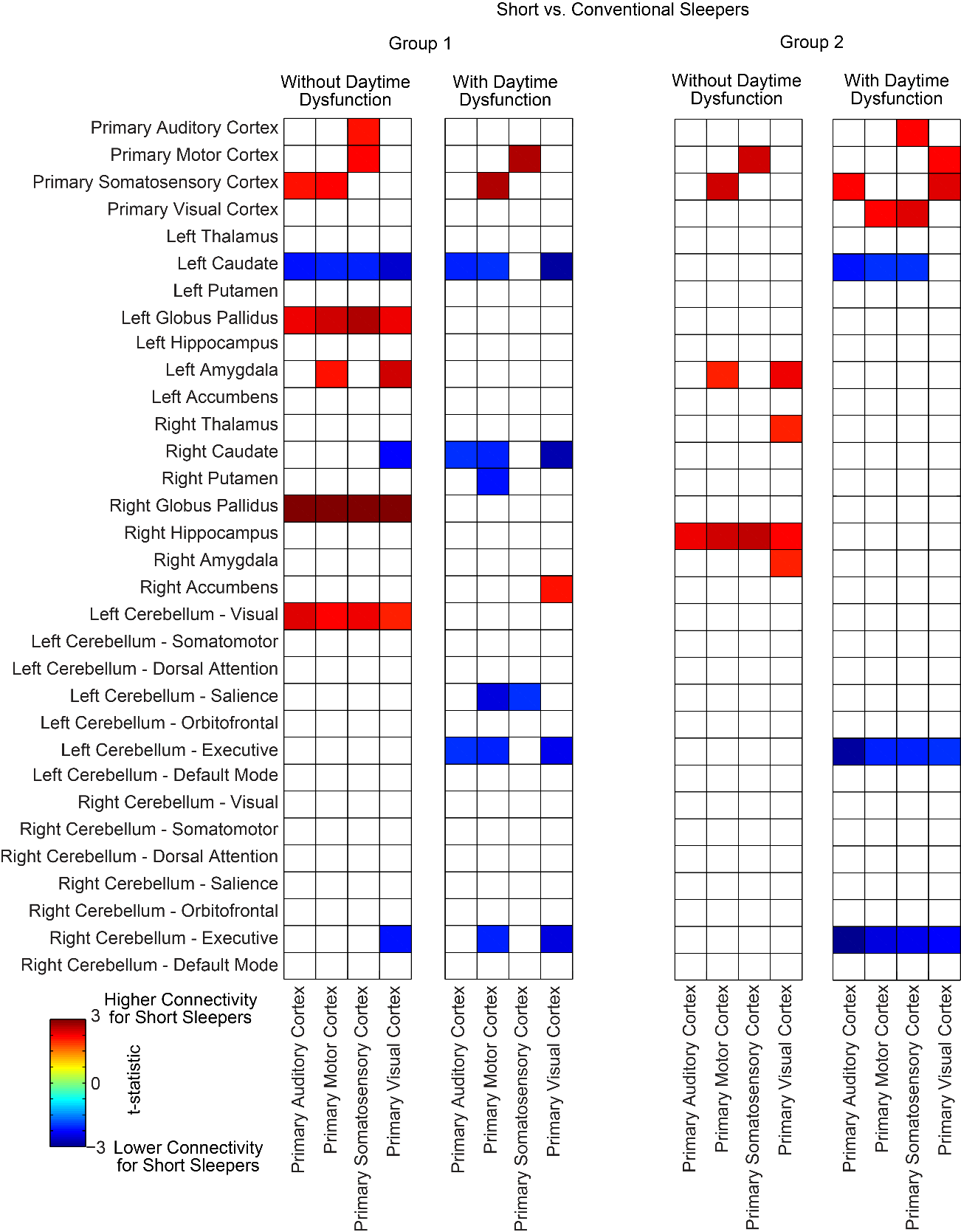


Figure S2: Differences in functional connectivity in self-reported short sleepers vs. conventional sleepers reporting and denying daytime dysfunction for each group. Colored squares satisfied p<0.05, uncorrected. Color scale represents t-statistic for a two-tailed t-test of functional connectivity between short and conventional sleepers.


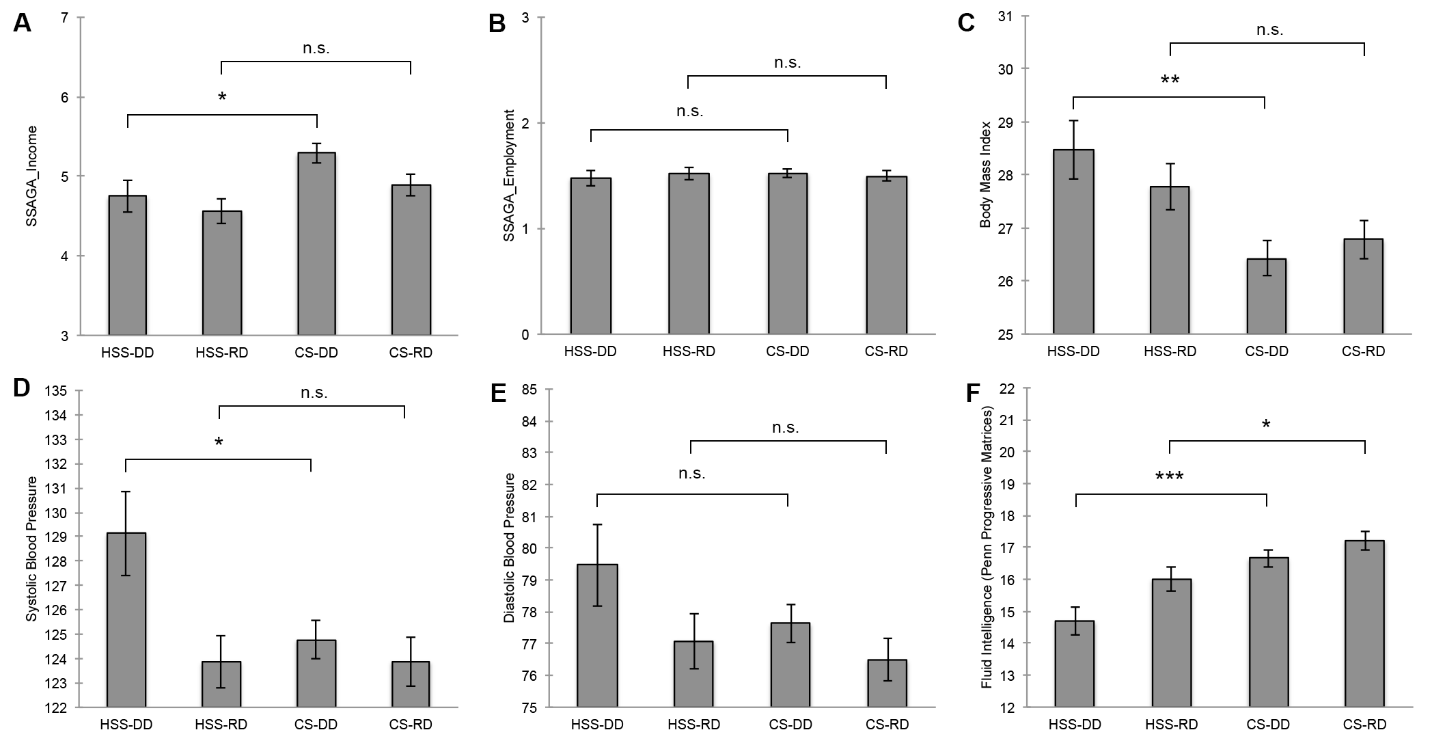


Figure S3. *Income, Employment, Body Mass Index, Blood Pressure, and Fluid Intelligence in Subgroups of Short and Conventional Sleepers*

Note: All analyses are independent samples two-tailed t-tests. Error bars represent standard error of the mean. SSAGA = Semi-Structured Assessment for the Genetics of Alcoholism. HSS-DD = habitual short sleepers denying daytime dysfunction. HSS-RD = habitual short sleepers reporting daytime dysfunction. CS-DD = conventional sleepers denying daytime dysfunction. CS-RD = conventional sleepers reporting daytime dysfunction.

*p < .05, **p < .01, ***p < .001
